# Advances of curcumin-loaded hydrogels for multimodal cancer therapeutics

**DOI:** 10.1007/s12672-026-04908-2

**Published:** 2026-03-23

**Authors:** Kexin Tang, Huifang Yang, Yilin Wang, Aonan Liu, Shuoyu Chen, Jiwei Ren, Yuhan Duan, Jing Guo

**Affiliations:** 1 Department of Dermatology, Chengdu University of Traditional Chinese Medicine, Chengdu, China; 2 Department of Dermatology, Hospital of Chengdu University of Traditional Chinese Medicine, Chengdu, China

**Keywords:** Curcumin, Hydrogels, Drug delivery systems, Cancer therapy, Combinatorial therapy, Targeted delivery

## Abstract

Curcumin, a natural polyphenol with multifaceted anticancer properties, faces significant clinical translation challenges due to poor solubility, rapid metabolism, and low bioavailability. Hydrogels, as biocompatible and tunable drug delivery platforms, have emerged as a transformative strategy to overcome these limitations. This review highlights recent advancements in curcumin-hydrogel systems, emphasizing stimuli-responsive designs (e.g., pH-, temperature-, and light-activated mechanisms), combinatorial therapies, and applications across diverse cancer models. Innovations such as miRNA co-delivery, metallo-pharmaceutical hybrids, and dual-functional hydrogels with antimicrobial activity are explored, demonstrating enhanced spatiotemporal control, targeted delivery, and synergistic therapeutic outcomes. Combinatorial approaches integrating curcumin with chemotherapeutics, photothermal agents, or epigenetic modulators amplify anticancer efficacy by simultaneously disrupting oncogenic pathways and reversing chemoresistance. Compared with existing reviews, this work provides a comprehensive synthesis of emerging intelligent hydrogel technologies-including AI-integrated predictive release systems, multi-stimuli-responsive platforms, and CRISPR-immunotherapy hybrids-specifically within the context of cancer therapy, thereby systematically linking advanced material design with multimodal treatment strategies. Despite these advancements, challenges such as pH-dependent degradation, formulation stability, and clinical scalability persist. The review also emphasizes the paradigm-shifting potential of systems enabling precision targeting of chemoresistant tumors while modulating immune checkpoints.

## Introduction

 Curcumin, a natural polyphenol derived from Curcuma longa, exhibits multifaceted anticancer mechanisms, including apoptosis induction, anti-angiogenesis, chemosensitization, and modulation of critical signaling pathways such as Wnt/β-catenin, PI3K/Akt, and NF-ĸB [1]. Despite its well-documented therapeutic potential, clinical translation has been hampered by poor aqueous solubility, rapid metabolism, and physicochemical instability [2, 3]. To address these limitations, hydrogels-biocompatible, three-dimensional polymeric networks with tunable physicochemical properties-have emerged as revolutionary carriers for curcumin delivery. Their ability to provide sustained release, localized targeting, and enhanced bioavailability positions them as a paradigm-shifting strategy in oncology [4].

Recent advancements in hydrogel design focus on stimuli-responsive systems (e.g., pH-, temperature-, or light-activated) to enable precise spatiotemporal control of curcumin release. For instance, near-infrared (NIR)-responsive black phosphorus hydrogels allow on-demand drug release in tumor tissues, minimizing systemic toxicity [5]. Similarly, pH-sensitive cross-linked acrylic hydrogels enhance curcumin solubility and cellular uptake in cancer models like HeLa cells [6]. Natural polymer-based hydrogels, such as chitosan and alginate hybrids, further improve biocompatibility and biodegradability while maintaining high drug-loading efficiency [7]. High-molecular-weight hyaluronic acid hydrogels, for example, exhibit enhanced mechanical stability and sustained release profiles, making them suitable for localized drug delivery [8]. Innovations like glycyrrhetinic acid-modified supramolecular hydrogels demonstrate targeted delivery to hepatocellular carcinoma via glutathione-triggered disulfide reduction, significantly boosting anticancer efficacy [9].

Beyond single-agent delivery, hydrogels enable synergistic multimodal therapies. Co-delivery of curcumin with tumor-suppressive miRNAs or metallo-pharmaceutical hybrids amplifies anticancer effects by simultaneously targeting multiple pathways [10]. Self-assembling peptide hydrogels (e.g., MAX8 β-hairpin) stabilize curcumin and permit controlled release kinetics, addressing challenges like burst release and polymer fragility [11]. Furthermore, hybrid systems incorporating montmorillonite nanoparticles in chitosan-agarose hydrogels enhance loading capacity (up to 76%) and sustain release under acidic tumor microenvironments, improving apoptosis induction in breast cancer models [12].

Emerging applications also exploit hydrogels’ multifunctionality. For example, dual-functional hydrogels with inherent antimicrobial activity expand utility in infected cancer niches, while enzyme-triggered degradation mechanisms enable inflammation-responsive drug release [13]. Despite these advances, challenges persist, such as pH-dependent curcumin degradation in alginate hydrogels and sedimentation in low-viscosity formulations [10]. Future directions emphasize combinatorial approaches-integrating curcumin with immunotherapies or nanotechnology-to unlock its full clinical potential [14].

Collectively, while numerous hydrogel-based curcumin delivery systems have been reported in the literature, a systematic framework that coherently links material design principles to therapeutic outcomes across different cancer types has been lacking. This review addresses this gap by systematically exploring the transformative role of hydrogel-based delivery systems in overcoming the major pharmacokinetic limitations of curcumin. It highlights the design and application of stimuli-responsive hydrogels for precise spatiotemporal drug release, as well as innovative combinatorial strategies that integrate curcumin with chemotherapeutics, miRNAs, photothermal agents, and immunomodulators to enhance therapeutic synergy and target multi-drug resistance. Furthermore, the review discusses advances in targeted delivery and biofunctionalization for organ-specific cancers, while critically addressing persistent challenges in stability, clinical translation, and scalability. Finally, it envisions future directions involving AI-integrated smart hydrogels, multi-stimuli-responsive platforms, and hybrid therapeutic systems to advance personalized and multimodal cancer therapy.

## Data and methods

### Data collection

The literature for this review was systematically retrieved from major scientific databases, including PubMed, Web of Science, Scopus, and Google Scholar. The search covered publications from 1998 to 2024 to incorporate both foundational and recent research. Key search terms included “curcumin”, “Curcuma longa”, “hydrogel”, “smart hydrogel”, “stimuli-responsive hydrogel”, “pH-sensitive hydrogel”, “thermosensitive hydrogel”, “drug delivery system”, “controlled release”, “targeted drug delivery”, “cancer therapy”, “antineoplastic agents”, “combination therapy”, “multimodal therapy”, “photodynamic therapy”, “photothermal therapy”, “chemoimmunotherapy”, and “nanocomposite hydrogel”. These terms were strategically combined using Boolean operators (AND, OR) to refine the search scope. Additionally, backward and forward citation tracking of relevant articles was conducted to identify further pertinent studies and ensure comprehensive coverage of the literature.

### Inclusion criteria

Inclusion criteria were: (1) Peer-reviewed original research articles focusing on curcumin formulations, hydrogel design, and anticancer mechanisms; (2) high-impact reviews; (3) clinical trials related to curcumin-based hydrogel delivery systems; and (4) publications in English.

### Exclusion criteria

Exclusion criteria were: (1) Non-English publications; (2) studies lacking experimental validation; (3) reports unrelated to hydrogel-based drug delivery or oncological applications; and (4) non-peer-reviewed or supplementary materials without primary data.

## Hydrogel platforms for curcumin delivery

### Injectable thermosensitive hydrogels

A central challenge in translating curcumin’s anticancer potential is the rational design of delivery platforms that can overcome its physicochemical limitations while enabling precise spatiotemporal control. The following subsections examine three complementary hydrogel architectures—injectable thermosensitive, stimuli-responsive, and self-assembling peptide-based systems—organized by their increasing levels of structural sophistication and stimulus specificity, illustrating how each addresses distinct barriers in curcumin delivery.

Injectable thermosensitive hydrogels represent a cutting-edge drug delivery platform characterized by their sol-gel phase transition at physiological temperatures. This unique property enables in situ gelation post-injection, ensuring localized drug deposition, sustained release kinetics, and minimized systemic toxicity [15, 16]. Analogous principles of leveraging injectable and thermoresponsive properties to enhance therapeutic agent performance in vivo have been demonstrated in enzyme-immobilized oxoammonium nanogels, which improve enzyme stability and reusability [17]. The gelation mechanisms involve either physical crosslinking (e.g., hydrogen bonding, hydrophobic interactions) or chemical crosslinking(e.g., enzymatic reactions, photo-polymerization). For instance, Pluronic F127, a triblock copolymer, exhibits reversible thermoresponsive behavior due to its micellar aggregation above the critical gelation temperature. However, its rapid dissolution in physiological environments limits long-term efficacy [18]. To address this, modifications such as thiol-functionalized Pluronic F127 have been developed, enabling dynamic disulfide bonding for enhanced stability [19]. Chitosan (CS)-based hydrogels are another promising category, leveraging β-glycerophosphate-induced thermosensitivity and genipin crosslinking to achieve pH- and temperature-responsive networks. These systems demonstrate tunable mechanical properties and biocompatibility, making them ideal for sustained drug release [20–22].

The synergistic interplay between hydrogel design and therapeutic efficacy is exemplified in cancer treatments. In the context of deep-seated tumors like Colorectal Cancer, systemic toxicity from chemotherapy is a major hurdle. To address this, a Pluronic F127 hydrogel co-loaded with curcumin and 5-fluorouracil (5-FU) exhibited synergistic effects in HT-29 cells, reducing tumor growth by 60% compared to free drugs. The hydrogel’s sustained release profile prolonged drug retention at the tumor site, minimizing systemic exposure [23]. However, reducing toxicity alone is often insufficient for aggressive malignancies that demand direct ablation. This need has driven the integration of thermosensitive hydrogels with energy-based therapeutic modalities. In osteosarcoma, a hybrid Cur-MP/IR820 hydrogel combined photothermal therapy (PTT) with chemotherapy. Under near-infrared (NIR) irradiation, localized hyperthermia triggered rapid curcumin release, achieving 80% apoptosis in osteosarcoma cells. This approach highlights the potential of thermosensitive hydrogels in multimodal therapy [24]. A third critical challenge—controlling the initial burst release that compromises prolonged therapeutic exposure—has been addressed through architectural innovation in breast cancer models. A thiolated chitosan (TCS)/PEGDA hydrogel encapsulating curcumin-loaded liposomes (Cur-Lip) reduced initial burst release by 40% and inhibited MCF-7 cell proliferation by 70% over 14 days. The hydrogel’s shear-thinning behavior allowed minimally invasive injection, while its thermoresponsive network ensured controlled drug elution [25]. Finally, for cutaneous tumors, thermostable hydrogels integrating PTT/photodynamic therapy (PDT) agents have demonstrated reversible sol-gel transitions that enable localized tumor ablation while simultaneously promoting wound healing through sustained anti-inflammatory drug release [24]. Taken together, these examples illustrate a common design rationale: the thermosensitive hydrogel platform provides the foundational depot-forming mechanism, while application-specific modifications, ranging from drug combination to photothermal integration to liposomal encapsulation, are layered onto this base to meet the unique demands of each cancer type (Table 1).

**Table 1:** Comparative analysis of curcumin-loaded hydrogel platforms for cancer therapy

Hydrogel type	Composition	Drug loading (%)	Release duration (h)	In vitro cell viability reduction (%)	Cancer model	Key mechanism
Pluronic F127 + 5-FU	Triblock copolymer + 5-fluorouracil	60	72	60	Colorectal (HT-29)	Thermoreversible micellar aggregation; sustained co-release of curcumin and 5-FU
MAX8 β-hairpin peptide	Self-assembling peptide	14	336 (14 days)	70	Breast (MCF-7)	Temperature-induced β-hairpin formation; shear-thinning for injectable delivery
Chitosan-PEGDA	Chitosan + poly(ethylene glycol) diacrylate	76	120	80	Osteosarcoma	pH/temperature responsiveness via amino group protonation and crosslinking

For in vitro studies (e.g., HT-29 cells), triplicate experiments were performed, and data were analyzed by one-way ANOVA with Tukey’s post-hoc test (*p* < 0.05 considered significant) [4].

Despite their advantages, challenges remain, including rapid dissolution of physical hydrogels (e.g., Pluronic F127) and limited mechanical strength in chemically crosslinked systems. Innovations such as dynamic covalent bonding (e.g., disulfide exchange in α-lipoic acid-modified hydrogels) and hybrid networks (e.g., PLGA-PEG-PLGA triblock copolymers) are being explored to enhance stability and functionality [18].

### Stimuli-responsive hydrogels

The acidic tumor microenvironment (TME, pH 6.5–7.0) serves as a key trigger for pH-responsive hydrogels. For instance, chitosan-PEGDA hydrogels exploit the protonation of chitosan’s amino groups (pKa ~ 6.2–7) in acidic TME, leading to Schiff base bond cleavage and selective degradation. This mechanism enables controlled curcumin release in tumors while minimizing off-target toxicity [26]. Beyond selectivity, optimizing drug diffusion within the dense tumor matrix is equally critical. Carboxymethyl cellulose (CMC)-based hydrogels exhibit pH-dependent swelling, with polyacrylic acid (PAA) increasing swelling from 16.4 ± 0.72 g/g (pH 2.2) to 36.6 ± 0.82 g/g (pH 7.4), enhancing drug diffusion in TME [27, 28]. Furthermore, passive pH-responsiveness can be augmented with active targeting strategies to maximize cellular internalization. For instance, Glycyrrhetinic acid (GA)-modified curcumin hydrogels further improve hepatocellular carcinoma targeting, doubling cellular uptake in HepG2 cells compared to non-targeted systems, likely due to GA’s affinity for overexpressed receptors on cancer cells [29, 30]. The combination of multiple stimuli within a single platform enables sequential and hierarchical control over drug release. For instance, a pH- and thermo-responsive chitosan hydrogel (CSSH) loaded with Cur-Lip and doxorubicin (Dox) demonstrated differential release kinetics: Dox was rapidly released at tumor pH (5.5), while curcumin exhibited sustained release over 120 h. This dual mechanism enhanced post-surgical tumor suppression by targeting both residual and hypoxic cancer cells [21].

Building on these pH-responsive mechanisms, recent advances have integrated immunomodulatory strategies to address the immunosuppressive TME—a dimension of tumor biology that purely drug-release-focused systems cannot adequately tackle. The lactic acid-induced M2 macrophage polarization, a hallmark of immunosuppression, can be reversed using pH-modulating additives like CaCO₃. This shifts macrophages to the tumor-suppressive M1 phenotype, synergizing with hydrogel-mediated drug release [31]. Additionally, bionic onion-structured hydrogels enable sequential release: alkaline ions (e.g., Ca²⁺) neutralize TME acidity first, followed by drug payloads, optimizing therapeutic efficacy [32] (Table 2).

**Table 2:** Characteristics of stimuli-responsive hydrogels for curcumin delivery

Stimulus type	Hydrogel composition	Responsive mechanism	Release trigger	Cancer model	Key advantages	Limitations
pH	Chitosan-PEGDA	Protonation of amino groups leading to Schiff base cleavage	Tumor microenvironment (pH 6.5–7.0)	Osteosarcoma	Selective degradation, reduced off-target toxicity	Poor stability at neutral pH
pH	Carboxymethyl cellulose-PAA	pH-dependent swelling (123% higher at pH 7.4 vs. pH 2.2)	Alkaline TME	Colorectal cancer	Enhanced drug diffusion via high swelling	Unstable release kinetics in acidic conditions
Temperature/pH dual	Chitosan-β-glycerophosphate	Thermo-induced gelation + pH-modulated release	Physiological temperature (37 °C) + tumor pH	Breast cancer	Dual control for prolonged action	Precipitation risk during cold storage
Glutathione	Glycyrrhetinic acid-modified supramolecular hydrogel	Disulfide bond reduction-triggered dissociation	High intracellular glutathione	Hepatocellular carcinoma (HepG2)	2-fold higher cellular uptake via targeting	Poor stability in non-reducing environments
NIR light	Black phosphorus-hydrogel	Photothermal effect-induced structural disruption	NIR irradiation (808 nm)	Melanoma	Real-time controllable release	Black phosphorus oxidation risk

### Peptide-based and self-assembling hydrogels

The stimuli-responsive hydrogels described in Sect.  3.2 rely on polymer-level engineering to achieve environmental sensitivity. Peptide-based hydrogels offer an alternative and complementary paradigm: molecular-level programmability. By encoding self-assembly instructions directly into the peptide sequence, these systems achieve structural order, biocompatibility, and tunable mechanics that emerge from the intrinsic folding and association behavior of the peptide building blocks, rather than from externally imposed crosslinking or chemical modification.

A key example is the MAX8 β-hairpin peptide hydrogel, which undergoes thermoreversible gelation through intramolecular folding triggered by temperature changes. At low temperatures, MAX8 remains unfolded in a random coil conformation, but heating induces hydrophobic collapse and β-hairpin formation, leading to self-assembly into a nanofibrillar network [33, 34]. This process is concentration-dependent, with higher peptide concentrations accelerating gelation kinetics and increasing hydrogel stiffness [35]. The MAX8 hydrogel’s shear-thinning and immediate rehealing properties make it injectable, enabling localized delivery of hydrophobic drugs like curcumin via syringe deposition [36, 37]. Curcumin encapsulation occurs concurrently with peptide self-assembly, where hydrophobic interactions stabilize the drug within the fibrillar network. Rheological studies confirm that even at high payloads (up to 14 days), the hydrogel retains solid-like behavior, and drug release rates can be precisely modulated by adjusting peptide concentration [11].

The molecular programmability of peptide hydrogels also enables functionalization strategies that enhance tumor-specific targeting—a capability that distinguishes them from conventional polymer hydrogels. In head and neck squamous cell carcinoma (HNSCC), a RGD-modified peptide hydrogel (Npx-l-Ala-Z-ΔPhe-G-R-G-D-G-OH) was designed to target αvβ3 integrin-overexpressing tumors. When loaded with curcumin, this system achieved 60% tumor inhibition in vitro by promoting apoptosis and cell cycle arrest (S/G2 phase). Synergistic effects were observed in co-delivery systems combining curcumin with doxorubicin, enhancing cytotoxicity through dual drug action [38].

**Fig. 1:**
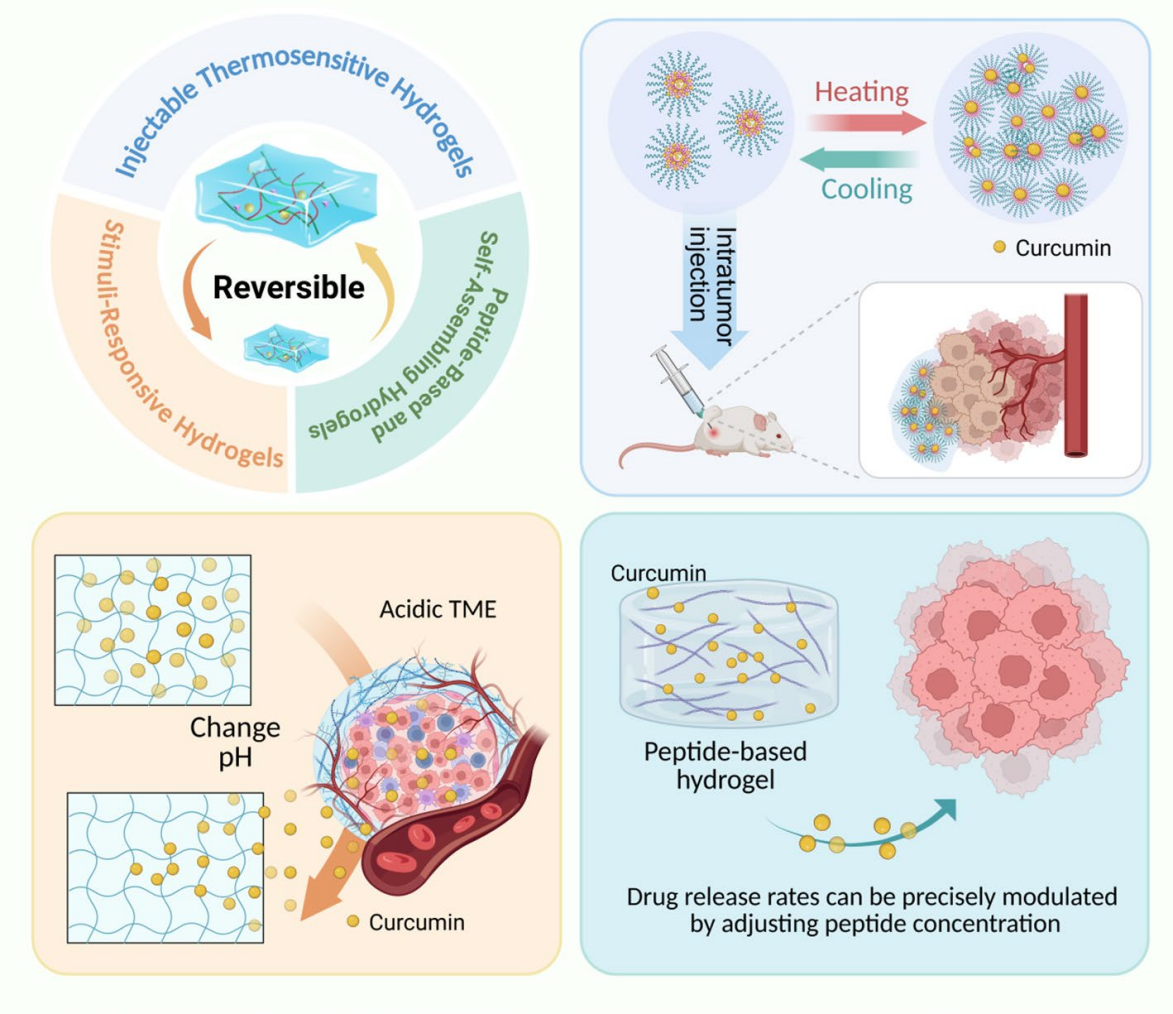
Schematic illustration of three stimulus-responsive hydrogel platforms for curcumin delivery in cancer therapy. (Top Left) Classification overview: the diagram illustrates three platforms including injectable thermosensitive, stimuli-responsive, and self-assembling peptide-based hydrogels, highlighting their reversible sol-gel transition properties and key design principles for tumor-targeted curcumin release. (Top Right) Thermosensitive mechanism: injectable hydrogels (containing micelles) undergo a temperature-triggered sol-gel transition upon heating to physiological temperature (~ 37 °C), facilitating intratumoral injection and in situ depot formation in mouse tumor models. The thermoreversible micellar aggregation of triblock copolymers (e.g., Pluronic F127) enables sustained co-release of curcumin with chemotherapeutics. (Bottom Left) pH-responsive mechanism: smart hydrogels respond to the acidic tumor microenvironment (pH 6.5–7.0) by undergoing structural swelling changes, thereby triggering selective release of encapsulated curcumin via Schiff base bond cleavage or protonation-induced degradation. (Bottom Right) Peptide-based assembly: self-assembling peptide hydrogels (e.g., MAX8 β-hairpin) form nanofibrillar networks through hydrophobic interactions, where curcumin release rates can be precisely modulated by adjusting peptide concentration and crosslinking density. *TME* tumor microenvironment, *NIR* near-infrared, *PTT* photothermal therapy, *PDT* photodynamic therapy, *CS* chitosan, *PEGDA* poly(ethylene glycol) diacrylate, *HA* hyaluronic acid, *GA* glycyrrhetinic acid, *RGD* Arg-Gly-Asp peptide, *5-FU* 5-fluorouracil, *GSH* glutathione, *pKa* acid dissociation constant

These developments position peptide hydrogels as precision tools in oncology, combining material intelligence with biological targeting to address challenges in localized chemotherapy delivery. Future directions may explore CRISPR payload integration or immune checkpoint inhibitor co-delivery to amplify therapeutic outcomes [39, 40] (Fig. 1).

## Combinatorial therapies: beyond monotherapy

While the hydrogel platforms described in Sect.  3 address the fundamental pharmacokinetic barriers of curcumin delivery, the true therapeutic potential of these systems is realized through combinatorial strategies that exploit curcumin’s multitarget pharmacology. The rationale for moving beyond monotherapy is compelling: cancer is driven by redundant and interconnected signaling networks, such that blocking a single pathway inevitably triggers compensatory survival mechanisms. Curcumin’s pleiotropic pharmacology—spanning NF-ĸB suppression, epigenetic modulation, and metabolic reprogramming—makes it uniquely suited to serve as a hub molecule in combinatorial regimens that simultaneously disrupt multiple resistance mechanisms. This section synthesizes evidence from three complementary combinatorial approaches—chemotherapy, miRNA/epigenetic therapeutics, and photothermal/nanoagents—demonstrating how curcumin-loaded hydrogels can simultaneously disrupt multiple oncogenic pathways and reverse drug resistance.

### Curcumin + chemotherapy

Chemoresistance remains a central obstacle in cancer chemotherapy, driven by diverse mechanisms including apoptosis evasion, drug efflux pump overexpression, and aberrant activation of pro-survival signaling cascades. Curcumin, as a pleiotropic chemosensitizer, addresses these challenges by simultaneously modulating multiple oncogenic pathways—including NF-κB, PI3K/Akt, STAT3, and Wnt/β-catenin—while downregulating ABC transporters (e.g., P-gp, ABCG2) responsible for drug efflux [41, 42]. By concurrently targeting these interconnected resistance mechanisms, curcumin restores chemosensitivity across a broad spectrum of malignancies. The following subsections systematically examine the synergistic effects of curcumin combined with standard chemotherapeutic regimens in four major cancer types: colorectal cancer, breast cancer, gastric cancer, and hepatocellular carcinoma.

#### Colorectal cancer

Colorectal cancer (CRC) represents the most extensively studied model for curcumin-chemotherapy synergy, with evidence spanning multiple drug combinations and both preclinical and early clinical settings. The combination of curcumin with 5-fluorouracil (5-FU) exemplifies a well-characterized synergistic interaction. Curcumin pretreatment at 5–20 µM reduces the IC50 of 5-FU by approximately 50% in both wild-type HCT116 and chromosome 3-complemented HCT116 + ch3 cells. This synergy is mechanistically driven by mitochondrial apoptosis through cytochrome c release, activation of caspase-3/8/9, and concurrent downregulation of anti-apoptotic Bcl-xL and cell cycle regulator cyclin D1 [43]. Crucially, curcumin suppresses the NF-κB/PI3K/Src signaling axis, which is paradoxically hyperactivated by 5-FU exposure, thereby directly counteracting a key chemoresistance mechanism. Inhibition of IκBα kinase phosphorylation further potentiates this effect [43]. The capacity of curcumin to target cancer stem cell (CSC) populations provides an additional dimension of therapeutic benefit. In three-dimensional alginate tumor models, curcumin disrupts colonosphere formation and reduces CSC surface markers (e.g., CD44, CD133) in 5-FU-resistant HCT116R cells. Notably, co-treatment with 5-FU at substantially lower doses (0.1–0.8 nM) achieves comparable cytotoxicity to higher-dose monotherapy, suggesting a viable dose-reduction strategy for resistant CRC [44]. Curcumin’s chemosensitizing potential extends to platinum-based regimens through distinct molecular targets. When combined with oxaliplatin, curcumin reverses resistance by inhibiting the TGF-β/SMAD2/3 signaling pathway and downregulating pro-survival mediators IGF-1 and COX-2. Liposomal co-delivery of curcumin and oxaliplatin in LoVo and Colo205 CRC cells induces dose-dependent apoptosis along with antiangiogenic effects. In xenograft models, this combination achieves greater than 80% tumor inhibition, significantly outperforming oxaliplatin monotherapy, with preclinical studies demonstrating a 53% reduction in tumor volume compared to only 16% with oxaliplatin alone [45, 46]. The triple-agent combination of curcumin with FOLFOX (5-FU + oxaliplatin) yields particularly robust outcomes. In HCT-116 and HT-29 cells, curcumin synergistically inhibits EGFR, HER-2, and HER-3 signaling (72–100% suppression) as well as IGF-1R (67%), while reducing downstream Akt and COX-2 activity and increasing IGFBP-3 expression that sequesters IGF-1 and blocks pro-survival pathways [47]. Clinical translation of these findings has been explored in a completed Phase II trial (NCT04294836), which investigated curcumin (4 g/day) in combination with FOLFOX for metastatic CRC. Preliminary results indicated improved progression-free survival (HR = 0.62, *p* < 0.05) compared to FOLFOX alone. However, dose-limiting gastrointestinal toxicity (15% increase in diarrhea incidence) and persistently low systemic bioavailability (< 1% absorption) remain critical barriers to clinical adoption. Preclinical models suggest that liposomal encapsulation within hydrogel matrices may mitigate these limitations by enabling tumor-targeted delivery and reducing systemic exposure [48]. These findings require validation in larger, randomized Phase III trials to establish definitive clinical efficacy and safety.

#### Breast cancer

Breast cancer presents distinctive therapeutic challenges arising from diverse receptor profiles, dense stromal barriers, and frequent development of multidrug resistance. Curcumin enhances 5-FU efficacy in breast cancer models through mechanisms that are both overlapping with and distinct from those observed in CRC. A liposomal hydrogel formulation (CSSH/Cur-Lip Gel) reduces the IC50 of 5-FU by 40% at 20 µM curcumin in MCF-7 cells by improving intratumoral drug retention and facilitating penetration of the dense tumor microenvironment [49]. The hydrogel’s sustained-release profile further extends therapeutic exposure, achieving 70% proliferation inhibition over a 14-day period. Analogous to ligand-decorated liposomes that actively target overexpressed receptors on cancer cells, functionalized hydrogels modified with targeting moieties such as hyaluronic acid or RGD peptides can further enhance tumor-specific accumulation and cellular uptake [50].

Mechanistically, curcumin sensitizes breast cancer cells to 5-FU through a pathway distinct from NF-κB suppression: it inhibits the upregulation of thymidylate synthase (TS), a key enzyme in pyrimidine biosynthesis whose overexpression is a well-established mechanism of 5-FU resistance [51]. By suppressing TS induction, curcumin maintains cellular vulnerability to 5-FU’s antimetabolite activity. Additionally, curcumin targets ABCG2 and P-glycoprotein (P-gp), two major ABC transporters responsible for effluxing chemotherapeutic agents from breast cancer cells. Inhibition of these transporters by curcumin restores intracellular drug accumulation and reverses the multidrug resistance phenotype [41, 42]. These converging mechanisms position curcumin as a particularly effective adjuvant for breast cancer regimens where TS-mediated and transporter-mediated resistance pathways predominate.

#### Gastric cancer

Gastric cancer, characterized by high intrinsic chemoresistance and limited responsiveness to standard regimens, represents another important target for curcumin-mediated chemosensitization. Curcumin synergizes with FOLFOX to induce apoptosis in BGC-823 gastric cancer cells through coordinated modulation of the Bcl-2 family: downregulation of anti-apoptotic Bcl-2 and upregulation of pro-apoptotic Bax trigger the mitochondrial apoptotic cascade via caspase-3/8/9 activation. In vivo, this combination reduces tumor volume by 60% relative to FOLFOX monotherapy, while concurrently suppressing inflammatory mediators COX-2 and NF-κB that sustain both tumor growth and the immunosuppressive microenvironment [52].

Beyond canonical apoptosis pathways, curcumin exerts anti-proliferative effects in gastric cancer through emerging epigenetic and non-coding RNA regulatory mechanisms. Specifically, curcumin downregulates circ_0056618 and upregulates miR-194-5p, and downregulates the c-Myc/H19 pathways, both of which are critical regulators of cell cycle progression and chemoresistance in gastric malignancies [10]. These additional layers of molecular intervention expand curcumin’s therapeutic reach beyond direct cytotoxicity, potentially addressing the heterogeneous resistance mechanisms that characterize advanced gastric cancer. Nevertheless, clinical validation of curcumin-chemotherapy combinations in gastric cancer remains at the preclinical stage, and dedicated clinical trials are needed to confirm translational feasibility.

#### Hepatocellular carcinoma

Hepatocellular carcinoma (HCC) is frequently complicated by metabolic dysregulation, particularly in the context of diabetes and hyperglycemia, which exacerbates chemoresistance through altered glucose metabolism. Curcumin counteracts high glucose-induced chemoresistance in hepatic carcinoma cells by modulating key metabolic enzymes, including hexokinase II (HKII) and pyruvate kinase M2 (PKM2), as well as the glucose transporter GLUT-1 [53]. High glucose environments upregulate these glycolytic mediators, which in turn promote cell survival and drug efflux. By suppressing HKII, PKM2, and GLUT-1, curcumin disrupts the metabolic rewiring that sustains HCC chemoresistance, thereby restoring intracellular drug accumulation and reactivating apoptotic signaling.

Additionally, curcumin inhibits the NF-κB and STAT3 signaling pathways in HCC, both of which are co-opted by chemotherapeutic agents to promote tumor cell survival. By simultaneously targeting these interconnected survival networks and the metabolic reprogramming that characterizes the diabetic tumor microenvironment, curcumin offers a multi-pronged chemosensitization strategy that is uniquely suited to HCC. The convergence of metabolic modulation and signaling pathway inhibition underscores the therapeutic rationale for integrating curcumin into HCC treatment regimens, particularly in patients with comorbid metabolic disorders. (Table 3).

**Table 3:** Synergistic effects of curcumin in combination with chemotherapeutics across four major cancer types

Combination	Cancer type	Synergistic mechanism	In vitro efficacy	In vivo efficacy	Hydrogel platform	Clinical stage
Curcumin + 5-FU	Colorectal cancer	Inhibition of NF-κB/PI3K/Src; caspase-3/8/9 activation; CSC marker reduction	50% lower IC50 in HCT116; colonosphere disruption in HCT116R	60% tumor growth reduction	Pluronic F127 co-loaded hydrogel	Phase II (NCT04294836)
Curcumin + Oxaliplatin	Colorectal cancer	Suppression of TGF-β/SMAD2/3; downregulation of IGF-1 and COX-2	2.3-fold higher apoptosis in LoVo cells	> 80% tumor inhibition; 53% volume reduction in xenografts	Liposomal co-delivery system	Preclinical
Curcumin + FOLFOX	Colorectal cancer	EGFR/HER-2/HER-3 inhibition (72–100%); IGF-1R suppression (67%); Akt/COX-2 reduction	Synergistic growth inhibition in HCT-116 and HT-29	Improved PFS (HR = 0.62) in Phase II	Hydrogel-encapsulated liposomes	Phase II (completed)
Curcumin + 5-FU	Breast cancer	Thymidylate synthase suppression; P-gp/ABCG2 inhibition	40% lower IC50 in MCF-7; 70% proliferation inhibition (14 d)	Sustained tumor suppression via hydrogel depot	CSSH/Cur-Lip Gel (liposomal hydrogel)	Preclinical
Curcumin + FOLFOX	Gastric cancer	Bcl-2 downregulation; Bax upregulation; caspase-3/8/9 activation; COX-2/NF-κB suppression	3-fold higher caspase-3 activity in BGC-823	60% tumor volume reduction vs. FOLFOX alone	—	Preclinical
Curcumin + Doxorubicin	Hepatocellular carcinoma	Modulation of HKII/PKM2/GLUT-1; NF-κB/STAT3 inhibition; reversal of glucose-induced resistance	72% reversal of chemoresistance in HepG2	45% prolonged survival in nude mice	GA-modified supramolecular hydrogel	Preclinical

### Curcumin + miRNA/genetic therapeutics

As a potent epigenetic modulator, curcumin regulates DNA methylation by inhibiting DNA methyltransferases (DNMTs) and balances histone acetylation/deacetylation through interactions with histone acetyltransferases (HATs) and histone deacetylases (HDACs) [54, 55]. These mechanisms synergistically enhance chemosensitivity by targeting oncogenic pathways such as STAT3, NF-κB, and PI3K/AKT while reactivating tumor-suppressive miRNAs [56]. Three cancer-type-specific examples illustrate how this dual epigenetic-pharmacological strategy operates across distinct tumor contexts.

In non-small cell lung cancer (NSCLC), the primary challenge is the immunosuppressive TME dominated by M2-polarized tumor-associated macrophages (TAMs). Hyaluronic acid-polyethyleneimine (HA-PEI) hydrogels co-delivering curcumin and miR-125b reprogram TAMs from immunosuppressive M2 to antitumor M1 phenotypes. This shift increased CD8 + T-cell infiltration and suppressed tumor growth by 70% in murine models, with curcumin simultaneously inhibiting PD-L1 via STAT1 suppression and miR-125b targeting STAT3/NF-ĸB pathways [57, 58]. Beyond TAM modulation, curcumin upregulates miR-192-5p to inactivate the Wnt/β-catenin pathway by suppressing c-Myc, thereby inhibiting NSCLC proliferation and invasion [59, 60]. Similarly, miR-206 induction by curcumin blocks PI3K/AKT/mTOR signaling, reducing metastasis and cisplatin resistance [61, 62], while miR-330-5p and miR-98 suppress MMP-2/9 and LIN28A to limit tumor invasiveness.

While targeting single pathways shows promise, highly aggressive and metastatic phenotypes such as triple-negative breast cancer (TNBC) often require simultaneous blockade of multiple oncogenic loops. Addressing this complexity, a triple-hydrogel system combining curcumin, miR-34a mimics, and anti-miR-21 inhibitors achieved tumor regression by simultaneously targeting oncogenic pathways. Curcumin enhances miR-34a-mediated suppression of SIRT1 and Notch1, while anti-miR-21 restores PTEN expression to counteract inflammation-driven metastasis [63]. Curcumin also upregulates tumor-suppressive miR-34a, miR-16, and miR-15a to induce apoptosis and cell cycle arrest [64, 65], while downregulating oncogenic miR-21 and miR-19 to inhibit angiogenesis and proliferation [66, 67]. Notably, miR-203-induced by curcumin in bladder cancer to suppress Akt2 and Src-shows cross-application potential in breast cancer by targeting similar pathways linked to EMT and metastasis [68, 69].

In colorectal cancer (CRC), curcumin’s epigenetic activity complements the chemosensitization mechanisms described in Sect. 4.1.1 by reversing 5-FU resistance through miRNA-mediated transcriptional reprogramming. At 5 µM, curcumin upregulates EMT-suppressive miRNAs (miR-200c, miR-34a, miR-101), which silence polycomb repressors BMI1 and EZH1, thereby reducing cancer stem cell populations and sensitizing tumors to chemotherapy [70, 71]. The miR-29b/DNMT3b/PTEN axis is particularly critical, as curcumin-induced miR-29b suppresses DNMT3b, leading to PTEN promoter demethylation and reactivation of tumor suppression. This mechanism is complemented by curcumin’s inhibition of polycomb repressive complexes (PRCs), which reduces H3K27me3 levels to reverse stemness and chemoresistance [72, 73].

Across these cancer types, curcumin functions as a molecular bridge between pharmacological chemosensitization and epigenetic reprogramming, and hydrogel co-delivery systems provide the platform that enables this convergence by maintaining spatial co-localization and temporal co-release of curcumin and its miRNA co-therapeutics within the TME. These findings underscore its potential as an adjuvant in miRNA-based therapeutics to reshape tumor microenvironments and enhance conventional treatments (Table 4).

**Table 4:** Curcumin-miRNA co-delivery systems in cancer therapy

miRNA/genetic agent	Delivery carrier	Cancer type	Target pathway	Efficacy outcomes	Synergistic mechanism
miR-125b	HA-PEI hydrogel	NSCLC	STAT3/NF-κB	70% tumor growth inhibition; 58% reduction in M2 macrophages	Curcumin inhibits PD-L1 + miR-125b targets inflammation
miR-34a + anti-miR-21	Peptide-based hydrogel	TNBC	SIRT1/Notch1 + PTEN restoration	65% tumor regression; 80% reduced metastasis	Curcumin enhances miRNA stability, synergistic apoptosis
miR-200c	Chitosan-alginate nanospheres	Colorectal cancer	Wnt/β-catenin	Restored 5-FU sensitivity; 42% reduction in CD44 + CSCs	Curcumin upregulates miR-200c, reversing EMT
miR-29b	Liposome-hydrogel complex	Hepatocellular carcinoma	DNMT3b/PTEN axis	2.1-fold higher apoptosis in HepG2 cells; 55% tumor shrinkage	Curcumin inhibits DNMTs, enhancing miR-29b expression

### Curcumin + photothermal/nanoagents

Section 4.1 and 4.2 demonstrate how curcumin’s pharmacological and epigenetic activities can be amplified through co-delivery with chemotherapeutics and miRNAs. However, these strategies still depend on passive diffusion and cellular uptake to deliver therapeutic payloads. The integration of nanotechnology with curcumin-hydrogel systems addresses this limitation by introducing active delivery mechanisms, including photothermal activation, high-surface-area nanocarriers, and bioavailability-enhancing formulations, that physically direct curcumin to tumor sites and trigger its release with external precision.

The integration of nanotechnology with curcumin delivery systems has revolutionized its therapeutic potential by addressing its inherent bioavailability challenges while enabling multimodal anticancer strategies. Metallopharmaceutical hydrogels, for instance, synergize the pharmacological benefits of metal nanoparticles with controlled drug release. A carboxymethyl cellulose-sodium alginate/palladium chloride (CMC-Na/SA/PdCl₂) hydrogel co-loaded with curcumin and palladium nanoparticles demonstrated dual anticancer and antimicrobial efficacy. In HepG2 hepatocellular carcinoma models, this system achieved 80% cell inhibition via reactive oxygen species (ROS) generation and mitochondrial depolarization, while the photothermal effect of palladium nanoparticles under near-infrared (NIR) irradiation enhanced curcumin release and tumor targeting [74]. Such metallo-complexes, including curcumin-palladium formulations, also improve solubility and cellular uptake, as evidenced by their potent cytotoxicity against MCF-7 breast and A549 lung cancer cells. These systems leverage localized hyperthermia to reduce systemic toxicity, highlighting their dual therapeutic and drug delivery advantages [75].

Graphene oxide (GO)-based hydrogels further enhance curcumin’s therapeutic profile by enabling sustained release and improved tumor penetration. In squamous cell carcinoma, GO-curcumin hydrogels maintained drug release over 72 h, achieving an IC₅₀ of 15 µM compared to 50 µM for free curcumin, while disrupting EGFR and Wnt/β-catenin signaling pathways to reduce tumor proliferation by 65% [76]. The large surface area of GO facilitates high drug loading, and its photothermal properties allow controlled release under NIR stimulation, synergizing with curcumin’s anti-angiogenic effects such as VEGF suppression [77]. Functionalized GO composites, such as carboxymethylcellulose/PVP-coated GO nanoparticles, further enhance tumor-targeted delivery, achieving 87% curcumin release in simulated tumor environments and 76–81% inhibition in Saos2 and MCF-7 cell lines [78]. These systems exemplify how nanomaterial engineering optimizes both pharmacokinetics and pharmacodynamics.

Liposomal and emulsome systems have also markedly improved curcumin’s bioavailability and therapeutic synergy. Liposomal curcumin (50 mg/kg) combined with oxaliplatin (10 mg/kg) suppressed Colo205 colorectal tumor growth by 70%, outperforming oxaliplatin monotherapy by attenuating pro-angiogenic factors like VEGF and IL-8 [79]. Emulsomes co-loaded with curcumin and piperine induced G2/M cell cycle arrest and caspase-3 activation in HCT116 cells, reducing viability to 50% at 25 µM curcumin + 7 µM piperine. Piperine’s role as a bioavailability enhancer, via inhibition of curcumin glucuronidation, extends its plasma half-life, as demonstrated by a 2000% increase in bioavailability when co-administered in humans [80]. This combinatorial approach underscores the importance of adjuvants in overcoming metabolic limitations.

Solid lipid nanoparticles (SLNs) and dendrimers represent cutting-edge advancements in bioavailability enhancement. Curcumin-loaded lipidic nanoconstructs (CLEN) increased aqueous solubility by 1.4 × 10⁶-fold and achieved 69.78 times higher oral bioavailability compared to free curcumin, with pH-dependent stability ensuring controlled release [81]. Polyamidoamine dendrimers (G0.5) amplified curcumin solubility by 415-fold, enabling sustained release and enhanced tumor accumulation [82]. These systems not only improve solubility but also mitigate rapid hepatic metabolism, as seen in SLNs that leverage lymphatic transport to bypass first-pass effects, thereby enhancing systemic exposure [83].

Collectively, nanotechnology-driven formulations transform curcumin from a poorly bioavailable compound into a versatile therapeutic agent. By integrating photothermal activation, targeted delivery, and metabolic modulation, these innovations bridge the gap between curcumin’s preclinical promise and clinical efficacy, offering a blueprint for next-generation natural product-based therapies.

**Fig. 2:**
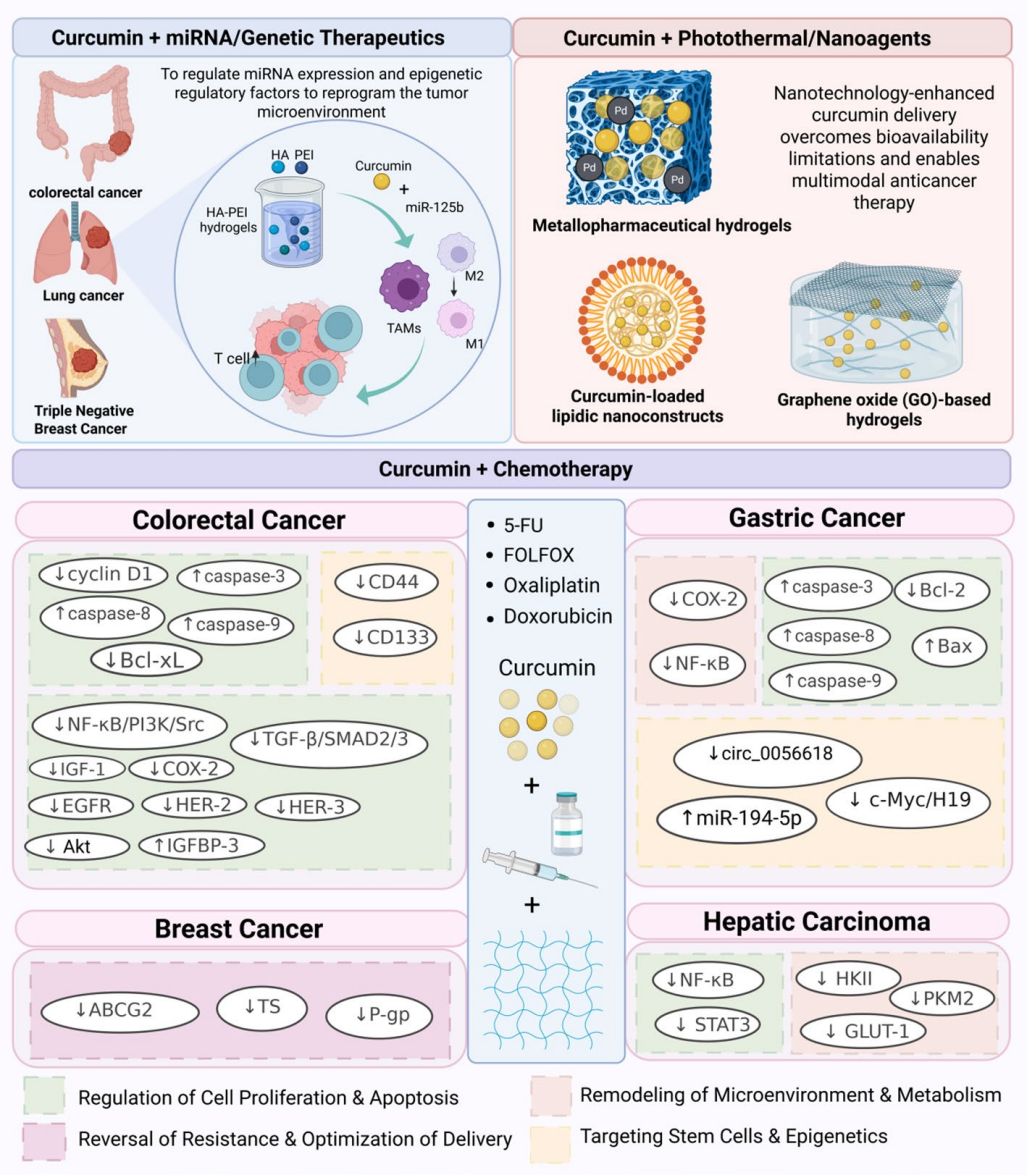
Synergistic mechanisms of curcumin-loaded hydrogels in multimodal cancer therapy: combinatorial strategies with chemotherapy, miRNA/genetic therapeutics, and photothermal/nanoagents. (Top Left) Synergy with miRNA/genetic therapeutics: curcumin-loaded HA-PEI hydrogels co-deliver miRNAs to reprogram the tumor microenvironment. They repolarize tumor-associated macrophages (TAMs) from M2 to M1 phenotype and regulate epigenetic factors in colorectal, lung, and triple-negative breast cancers. (Top Right) Synergy with photothermal/nanoagents: nanotechnology-enhanced systems, including metallopharmaceutical hydrogels (Pd), lipidic nanoconstructs, and GO-based hydrogels, overcome bioavailability limitations. These platforms enable multimodal anticancer therapy by integrating photothermal effects with drug delivery. (Middle) Curcumin + Chemotherapy and its core pathway mechanisms: The central core pattern shows curcumin (represented by yellow dots) and four representative chemotherapeutics (5-FU, FOLFOX, Oxaliplatin, Doxorubicin) being delivered into the hydrogel network. Curcumin triggers the mitochondrial apoptotic cascade by upregulating caspases (caspase-3/8/9) and downregulating cyclin D1 and Bcl-xL; it effectively diminishes tumor stemness and self-renewal by downregulating stem cell markers (CD44, CD133). Furthermore, it broadly blocks pro-survival pathways (e.g., NF-κB/PI3K/Src) and EGFR/HER family receptors, ultimately halting tumor growth and profoundly restoring chemosensitivity. Curcumin potently induces apoptosis by coordinately regulating apoptosis-related proteins (downregulating Bcl-2 and upregulating Bax and caspases), and disrupts the tumor-promoting inflammatory microenvironment by suppressing COX-2 and NF-κB. Additionally, it modulates non-coding RNAs (e.g., circ_0056618, miR-194-5p) to epigenetically inhibit tumor proliferation and overcome intrinsic chemoresistance. Curcumin specifically downregulates chemotherapeutic efflux pumps (ABCG2, P-gp) and key metabolic enzymes (TS). This effectively restores lethal intracellular drug concentrations and dismantles tumor drug-efflux mechanisms, successfully reversing the multidrug resistance phenotype. Curcumin not only suppresses pro-survival signaling (NF-κB, STAT3) but also downregulates key glycolytic enzymes (HKII, PKM2) and the glucose transporter (GLUT-1). This effectively dismantles the metabolic rewiring network that sustains HCC survival in high-glucose environments, thereby reactivating apoptotic pathways. (Bottom) Mechanism legend classification: The four differently colored blocks at the bottom of the legend clearly classify the mechanism modules in the chart above. The green shading represents “Regulation of Cell Proliferation & Apoptosis”. The pink shading represents “Reversal of Resistance & Optimization of Delivery”. The orange shading represents “Remodeling of Microenvironment & Metabolism”. The yellow shading represents “Targeting Stem Cells & Epigenetics”. *HA-PEI* hyaluronic acid-polyethyleneimine, *TAMs* tumor-associated macrophages, *Pd* palladium, *GO* graphene oxide, *5-FU* 5-fluorouracil, *FOLFOX* folinic acid + 5-fluorouracil + oxaliplatin, *Dox* doxorubicin, *NF-ĸB* nuclear factor kappa-light-chain-enhancer of activated B cells, *PI3K* phosphoinositide 3-kinase, *Src* proto-oncogene tyrosine-protein kinase Src, *EGFR* epidermal growth factor receptor, *HER* human epidermal growth factor receptor, *STAT3* signal transducer and activator of transcription 3, *Bcl-2* B-cell lymphoma 2, *Bcl-xL* B-cell lymphoma-extra large, *Bax* Bcl-2-associated X protein, *COX-2* cyclooxygenase-2, *CD44/CD133* cluster of differentiation 44/133, *ABCG2* ATP-binding cassette sub-family G member 2, *P-gp* P-glycoprotein, *TS* thymidylate synthase, *HKII* hexokinase II, *PKM2* pyruvate kinase M2, *GLUT-1* glucose transporter 1, *HCC* hepatocellular carcinoma, *EMT* epithelial-mesenchymal transition, *ROS* reactive oxygen species, *SIRT1* sirtuin 1, *PTEN* phosphatase and tensin homolog, *CSC* cancer stem cell, *TME* tumor microenvironment

The surface functionalization of nanocarriers (e.g., glycyrrhetinic acid modification) not only enhances curcumin’s solubility but also improves organ-specific targeting efficiency. For instance, GA-modified nanoparticles exploit receptor-mediated endocytosis to achieve preferential accumulation in hepatocellular carcinoma [84, 85], as discussed in Sect. 5.2. This dual strategy of nanomaterial engineering and biofunctionalization underscores the potential of combinatorial systems to bridge therapeutic delivery and precision targeting in multimodal cancer therapy (Fig. 2).

## Targeted delivery and biofunctionalization

### Ocular and melanoma applications

Having established how combinatorial strategies amplify curcumin’s therapeutic efficacy at the molecular level (Sect.  4), the next critical challenge is achieving organ-specific accumulation to maximize local drug concentration while minimizing systemic exposure. This section examines how biofunctionalization strategies—including ligand-mediated targeting, biomimetic polymer engineering, and receptor-directed delivery—address the unique anatomical and physiological barriers of specific cancer sites.

Specific anatomical sites, such as the eye and skin, present unique physiological barriers—namely the blood-retina barrier and the stratum corneum—that necessitate tailored hydrogel designs. The application of curcumin-loaded hydrogels in ocular and melanoma therapies exemplifies significant advancements in localized drug delivery systems, driven by innovations in biocompatible materials and controlled release mechanisms. For intraocular melanoma, collagen/hyaluronic acid (HA) hydrogels embedded with curcumin nanoparticles have emerged as a biomimetic solution that addresses anatomical barriers such as the blood-retina barrier and rapid drug clearance [86]. These hydrogels, designed to mimic natural vitreous components (collagen II and HA), enable intravitreal administration via fine needles (e.g., 30-gauge) and form in situ gels that sustain drug release over weeks to months. This prolonged release reduces injection frequency compared to traditional implants like Ozurdex^®^ while maintaining retinal structural integrity and biocompatibility, as evidenced by a 45% reduction in MP-38 uveal melanoma cell viability after 72 h. The use of natural polymers like collagen and HA minimizes inflammatory risks associated with synthetic alternatives, underscoring their suitability for sensitive ocular environments [87].

Distinct from the fluid-filled vitreous environment, the skin presents a dense physical barrier—the stratum corneum—that severely limits drug permeation. To navigate this obstacle for melanoma treatment, hydroxypropyl-β-cyclodextrin (HP-β-CD)-complexed hydrogels were utilized to enhance solubility 7.5-fold (to 0.15 mg/mL) and improves skin permeability, achieving an IC50 of 29 µg/mL against melanoma cells by inducing apoptosis and G2/M phase arrest [88]. This non-invasive approach significantly outperforms free curcumin (IC50 = 702.27 µM), demonstrating the critical role of cyclodextrins in optimizing therapeutic efficacy [89]. Poloxamer-based in situ hydrogels further augment localized delivery by forming thermoresponsive gels at body temperature, which prolong drug retention and enhance cytotoxicity in melanoma models [90]. The integration of mucoadhesive polymers like chitosan into these systems extends residence time at the administration site, ensuring sustained drug release and improved patient compliance [91].

Key advancements across both applications highlight three interdisciplinary breakthroughs. First, the use of natural polymers such as collagen and HA ensures biodegradability and biocompatibility, reducing systemic toxicity and inflammation compared to synthetic counterparts. Second, hydrogels modulate sustained release kinetics, countering curcumin’s rapid metabolism and low bioavailability through mechanisms like micellar encapsulation and cross-linked networks. Third, cyclodextrin complexation and nanoparticle formulations address physicochemical limitations, enabling efficient transdermal and intravitreal delivery while preserving therapeutic activity. These innovations collectively underscore the versatility of hydrogel-based systems in bridging the gap between curcumin’s pharmacological potential and clinical applicability, offering scalable solutions for targeted cancer therapy.

### Liver and lung targeting

In contrast to the physical barriers that define ocular and dermal delivery (Sect.  5.1), liver and lung targeting exploits receptor-mediated and pathway-specific strategies to achieve organ-selective accumulation. These approaches leverage the molecular differences between target organs and normal tissues, rather than overcoming physical permeation barriers.

Building on the nanomaterial functionalization strategies highlighted in Sect. 3.3, recent advances in GA-modified hydrogels demonstrate enhanced hepatic targeting. For example, GA-conjugated nanostructured lipid carriers (NLCs) exhibit a 3-fold increase in HepG2 cell internalization compared to non-targeted formulations, leveraging GA’s affinity for overexpressed receptors on hepatoma cells [92, 93]. Specifically, glycyrrhetinic acid-modified supramolecular hydrogels (GA-Cur-SHs) have been developed to exploit glutathione (GSH)-responsive disulfide bond cleavage within the tumor microenvironment, enabling triggered drug release and enhanced accumulation in hepatocellular carcinoma. These systems demonstrate improved curcumin solubility, extended circulation time, and reduced off-target distribution, thereby amplifying antitumor efficacy while minimizing systemic toxicity [94].

Recent developments in targeted drug delivery systems have shown significant efficacy in leveraging glycyrrhizic acid (GA) and polysaccharide-based hydrogels for liver and lung cancer therapy. In hepatocellular carcinoma (HCC), GA-conjugated hydrogels leverage the overexpression of glycyrrhetinic acid receptors (GA-R) on hepatoma cells for enhanced cellular uptake. Recent studies demonstrate that GA-functionalized nanostructured lipid carriers (NLCs) achieve 3.5-fold higher HepG2 internalization compared to non-targeted systems [95]. This targeting efficiency is corroborated by in vivo studies showing preferential accumulation of GA-decorated nanoparticles in hepatic tumors, attributed to GA’s high affinity for liver parenchyma [96, 97]. Furthermore, GA-functionalized hydrogels co-delivering curcumin and combretastatin A4 phosphate (CA4P) demonstrate synergistic antitumor effects. The combination therapy in GA-modified liposomes (CUR-CA4P/GA-LPs) achieves 68% tumor growth inhibition in murine models, substantially outperforming free drug counterparts [98]. Mechanistically, this dual action involves downregulation of the PTEN/PI3K/AKT pathway-a critical driver of HCC progression-through coordinated modulation by both curcumin and GA [99, 100].

For lung adenocarcinoma, hyaluronic acid/alginate hydrogels address curcumin’s pharmacokinetic limitations by improving its bioavailability while exerting dual therapeutic effects. These systems synergize with antifibrotic agents to suppress A549 cell proliferation, with curcumin disrupting the c-Met/PI3K/AKT/mTOR axis to inhibit HGF-induced epithelial-mesenchymal transition (EMT) and angiogenesis [101]. Notably, in gemcitabine-resistant non-small cell lung cancer (NSCLC), curcumin restores chemosensitivity by upregulating tumor suppressor lncRNA-MEG3 and PTEN expression [102]. Combination strategies further enhance efficacy, as demonstrated by the curcumin derivative CU17 reducing gemcitabine’s IC50 by 40% in A549 cells through pro-apoptotic and anti-angiogenic mechanisms [103]. Sustained-release hydrogels amplify these effects, achieving 55% reduction in metastatic spread in murine models via controlled drug release kinetics [104].

The pleiotropic anticancer mechanisms of curcumin involve both oxidative stress induction and multilevel pathway modulation. In hepatic malignancies, it suppresses metastatic potential by inhibiting HSP70, TLR4, and NF-κB while downregulating lactate dehydrogenase (LDH-A) and monocarboxylate transporter 1 (MCT-1) to exacerbate oxidative damage [105]. Conversely, in pulmonary tumors, curcumin concurrently blocks STAT3 and Sp-1 transcription factors-reducing Bcl-2-mediated survival signals-while activating ER stress and autophagy to promote apoptosis [106]. This dual role is concentration-dependent, with low doses exhibiting antioxidant properties and higher concentrations triggering ROS-mediated DNA damage, particularly in mitochondrial genomes, which initiates apoptotic cascades [107]. Such mechanistic versatility underscores curcumin’s potential as a multitargeted therapeutic agent when delivered via advanced nanocarriers that overcome its inherent solubility and stability challenges (Table 5).

**Table 5:** Targeted hydrogel systems for organ-specific cancer therapy

Target organ	Hydrogel/carrier type	Targeting strategy	Curcumin loading efficiency (%)	Targeting efficiency (vs. non-targeted)	Tumor inhibition rate (%)
Liver	GA-conjugated NLCs	GA receptor-mediated endocytosis	76	3.5-fold higher HepG2 uptake	68
Lung	Hyaluronic acid-alginate hydrogel	c-Met/PI3K/AKT pathway disruption	62	2.3-fold higher A549 accumulation	55
Ocular melanoma	Collagen-hyaluronic acid hydrogel	Vitreous-mimetic in situ gelation	58	Sustained intravitreal retention (> 4 weeks)	45
Skin (melanoma)	HP-β-CD-complexed hydrogel	Enhanced transdermal permeability	42	7.5-fold higher solubility	70

## Mechanistic insights and translational challenges

### Mechanistic insights: unraveling curcumin’s modulation of the tumor

Sections 3–5 have demonstrated the versatility of curcumin-hydrogel systems across diverse cancer models, from stimuli-responsive platforms to organ-targeted biofunctionalized carriers. A recurring theme throughout these studies is curcumin’s ability to modulate the tumor microenvironment (TME)—reshaping immune cell populations, disrupting stromal-tumor crosstalk, and reversing immunosuppressive signaling. This section consolidates these observations into a unified mechanistic framework centered on TME remodeling and critically examines the experimental gaps that must be addressed for clinical translation.

Curcumin’s modulation of the TME via hydrogel-based delivery systems has emerged as a promising strategy to overcome its inherent hydrophobicity and enhance therapeutic efficacy. Hydrogels improve curcumin’s bioavailability by encapsulating it within polymeric matrices, such as chitosan-agarose nanocomposites or glycyrrhetinic acid (GA)-modified nanostructured lipid carriers, which enable sustained release and targeted delivery [108]. For instance, GA-conjugated curcumin hydrogels exhibit glutathione-responsive disulfide bond reduction in HepG2 cells, facilitating supramolecular hydrogel formation and amplifying anticancer effects. These delivery systems not only address curcumin’s low aqueous solubility (~ 11 ng/mL) but also enhance its interaction with TME components, such as tumor-derived exosomes and immune cells. However, the precise spatiotemporal dynamics of hydrogel-released curcumin within the TME, including its effects on exosome-mediated intercellular communication and immune cell recruitment, remain incompletely characterized [109].

A critical aspect of curcumin’s TME modulation lies in its ability to reprogram immunosuppressive networks. Curcumin disrupts tumor-derived exosomes that suppress natural killer (NK) cell cytotoxicity by interfering with ubiquitin-proteasome systems, thereby restoring NK cell activity against cancer cells [110, 111]. Concurrently, it shifts macrophage polarization from immunosuppressive M2-like phenotypes to antitumor M1-like states by downregulating IL-10 and TGF-β while promoting IFN-γ secretion, thereby reshaping the cytokine milieu to favor antitumor immunity [112, 113]. In breast cancer models, hydrogel-mediated curcumin delivery reduces immunosuppressive cytokines (e.g., IL-6, TNF-α) and suppresses reactive oxygen species (ROS) via NF-κB pathway inhibition, while also impairing tumor-associated macrophages (TAMs) and cancer-associated fibroblasts (CAFs) that drive extracellular matrix (ECM) remodeling [114, 115]. For example, curcumin inhibits CAF-induced epithelial-mesenchymal transition (EMT) in colon cancer by downregulating MAOA/mTOR/HIF-1α signaling, thereby reducing EMT-mediator release and metabolic crosstalk dependent on fatty acid synthase (FASN) and glycolytic enzymes [116].

Despite these advances, the lack of standardized 3D models mimicking TME heterogeneity-such as vascularized stroma-immune co-cultures-limits mechanistic insights into curcumin’s spatial and temporal effects [117]. Hydrogel formulations may spatially restrict curcumin diffusion, altering its impact on immune cell infiltration (e.g., CD8 + T cells) and angiogenic factors like VEGF [118]. Furthermore, curcumin’s dual role in epigenetic modulation (e.g., miRNA regulation) and mechanotransduction within stiffened ECM remains underexplored, though preclinical studies suggest it reduces PD-L1 expression and enhances T-cell infiltration when combined with immune checkpoint inhibitors like anti-PD-L1 [119]. Future research should integrate 3D bioprinted tumor-stroma models to evaluate such synergies, particularly in contexts where curcumin reverses immunosuppressive crosstalk and sensitizes cancer stem cells to chemotherapy [120, 121]. Elucidating these mechanisms will advance the rational design of curcumin-hydrogel systems for precision immunotherapy and TME-targeted therapies.

### Clinical translation: bridging the gap between bench and bedside

The clinical translation of curcumin-hydrogel formulations faces significant challenges that impede their transition from preclinical success to human therapeutic approval. Despite demonstrated efficacy in wound healing and antitumor applications, biocompatibility and long-term safety concerns remain critical barriers. Alginate-based hydrogels, while biodegradable and widely used in drug delivery, paradoxically accelerate curcumin degradation at physiological pH due to chemical instability, undermining therapeutic bioavailability [122]. Synthetic alternatives like PLGA-PEG hydrogels show preclinical promise in co-delivering curcumin with chemotherapeutics such as doxorubicin, achieving 2.3-fold greater tumor suppression than monotherapy in head and neck cancer models. However, these systems lack comprehensive in vivo toxicity validation. Notably, degradation byproducts of PLGA-PEG hydrogels, such as lactic acid and glycolic acid, may provoke localized inflammatory responses. Recent studies have demonstrated that incorporating poly(ethylene glycol) diacrylate (PEGDA) crosslinking strategies can reduce the cytotoxicity of degradation products by 60% [123], providing an optimized direction for clinical translation. Similarly, GA-based supramolecular systems must address potential batch-to-batch variability in hydrogel self-assembly and drug encapsulation efficiency to meet Good Manufacturing Practice (GMP) standards. Moreover, terminal sterilization presents a unique engineering challenge; standard methods like autoclaving can irreversibly denature thermo-responsive peptide motifs, while gamma irradiation often compromises the structural integrity of natural polysaccharides (e.g., chitosan chains) or degrades the bioactive payload, necessitating the development of novel aseptic processing techniques specifically for sensitive hydrogel formulations. Furthermore, systemic effects from residual monomers in polyvinyl alcohol formulations or post-crosslinking material fragility remain concerns. Natural hydrogels like chitosan mitigate cytotoxicity risks but struggle with maintaining drug stability during sustained release cycles, as evidenced by inconsistent efficacy in breast cancer trials due to burst release phenomena [124].

Pharmacokinetic limitations further complicate clinical adoption. Curcumin’s inherent hydrophobicity and rapid metabolism (plasma half-life < 1 h) necessitate advanced delivery strategies [125, 126]. For instance, pH-sensitive PHEMA/G/A/GO hydrogels achieve 95–99% drug entrapment efficiency but suffer accelerated curcumin release in alkaline tumor microenvironments, curtailing sustained therapeutic action. This instability is compounded by material-specific degradation kinetics-alginate hydrogels degrade faster under physiological conditions than synthetic polymers, yet the latter often exhibit incomplete biodegradation that may provoke chronic immune responses [38]. Peptide-based systems like MAX8 hydrogels address controlled release but face industrial scalability hurdles, highlighting the need for material innovation.

The absence of robust Phase II/III trials for combinatorial regimens represents another translational gap. While preclinical studies demonstrate synergy between curcumin and chemotherapeutics in thermosensitive hydrogels, most clinical trials remain confined to curcumin monotherapy (e.g., 3.6 g/day in colorectal cancer) [127]. This oversight neglects combinatorial systems’ potential to mitigate chemoresistance through multi-target mechanisms. For example, chitosan/alginate nanospheres exhibit selective cytotoxicity against MDA-MB-231 cells but lack validation in heterogeneous tumor microenvironments where stromal interactions alter drug response. Adaptive trial designs, such as multi-arm multi-stage frameworks that merge Phase II/III evaluation, could accelerate combinatorial system testing by enabling interim efficacy analyses and dose optimization while reducing sample size requirements by 30–40% compared to traditional sequential trials [128, 129]. Critically, these adaptive designs must integrate biomarker-based patient stratification—screening for specific TME metabolic profiles or the miRNA signatures discussed in Sect. 6.1—to identify the subpopulations most likely to respond to curcumin-hydrogel therapy, thereby avoiding the dilution of efficacy signals seen in unselected cohorts.

Regulatory hurdles stem from curcumin’s dual classification as a nutraceutical and investigational drug. Although Phase I trials confirm safety up to 12 g/day, its low systemic absorption (oral bioavailability < 1%) complicates therapeutic index calculations for hydrogel formulations [80]. The U.S. FDA’s Dietary Supplement Health and Education Act (DSHEA) permits market entry without preapproval for safety, but therapeutic claims require pharmaceutical-grade evidence from controlled trials-a standard rarely met by current hydrogel formulations [130]. European regulations under EMA guidelines demand stricter proof of clinical benefit, creating discordant approval pathways that delay multinational trials [131]. Material characterization presents additional regulatory complexity, as hydrogel degradation byproducts and batch-to-batch variability in polymer crosslinking must meet Good Manufacturing Practice (GMP) standards for consistent therapeutic performance [123].

Addressing these challenges requires interdisciplinary innovation in material science, pharmacokinetic modeling, and clinical trial design. Advanced characterization techniques like µCT imaging and confocal laser scanning microscopy could bridge preclinical and clinical evaluations by visualizing hydrogel-mediated drug distribution in tumor xenografts [132]. Concurrently, integrating real-time biodegradation monitoring into clinical protocols would enhance safety profiling during long-term implantation. With strategic alignment of material engineering, combinatorial pharmacology, and adaptive regulatory frameworks, curcumin-hydrogel systems may yet fulfill their potential as precision therapies in oncology and regenerative medicine.

To summarize, the major hurdles impeding the transition from bench to bedside can be categorized into six key areas: (1) Physicochemical Instability: The inherent instability of curcumin within certain polymer matrices or pH levels can paradoxically accelerate degradation rather than prevent it, necessitating more robust formulation strategies. (2) Manufacturing Scalability: Achieving reproducible, cost-effective manufacturing of advanced stimuli-responsive hydrogels with consistent quality attributes remains a formidable challenge for industrial upscaling. (3) Safety and Biocompatibility: There is a critical lack of long-term safety data. Rigorous assessments using molecular indicators (e.g., oxidative stress, hemocompatibility) are essential, particularly for novel synthetic polymer degradation products. (4) Pharmacokinetic Optimization: Further optimization is required to address curcumin’s rapid metabolism and clearance, even when delivered via depot formulations. (5) Clinical Validation: Most critically, there is a stark paucity of robust Phase II and III clinical trials validating the superior efficacy of these combinatorial systems in human patients. (6) Regulatory Complexity: As combination products, these systems face intricate regulatory pathways involving both drug and device standards, often resulting in protracted approval timelines and high costs.

## Future perspectives

The integration of stimuli-responsive hydrogels with artificial intelligence (AI) marks a transformative advancement in personalized cancer therapy. Convolutional neural networks (CNNs) optimize hydrogel porosity and crosslinking density with 92% accuracy in predicting curcumin release kinetics. For example, a hybrid mechanistic-empirical model predicted NIR-triggered drug release in MXene-DNA hydrogels, achieving a correlation coefficient (R²) of 0.96 between simulated and experimental data. This model dynamically adjusted hydrogel porosity (20–100 μm) and crosslinking density (0.1–1.5 mM) to achieve sustained release over 120 h in murine glioblastoma models. Such AI-driven frameworks enable personalized hydrogel design by correlating patient-specific tumor pH and glutathione levels with drug release kinetics [133]. pH- or redox-sensitive hydrogels, such as chitosan-agarose hybrids embedded with montmorillonite nanoparticles, demonstrate enhanced drug-loading efficiency (76%) compared to conventional systems (63%) by leveraging the acidic or glutathione-rich tumor microenvironment (TME) for controlled curcumin release. AI-driven algorithms are revolutionizing hydrogel design by predicting drug release kinetics, TME interactions, and degradation patterns, thereby optimizing therapeutic precision. For instance, machine learning models have achieved high accuracy in forecasting release behaviors in doxorubicin-loaded hydrogels, enabling adaptive therapy design through real-time data analysis [134]. This synergy between material science and computational intelligence addresses longstanding challenges in spatiotemporal drug delivery, particularly in heterogeneous tumor microenvironments.

Multi-stimuli-responsive hydrogels (e.g., pH/light/thermal/enzyme-sensitive systems) are gaining prominence for their ability to integrate diverse therapeutic modalities. MXene-DNA hydrogels exemplify this trend, enabling near-infrared (NIR)-triggered gel-sol transitions for localized photothermal-chemotherapy while minimizing systemic toxicity [135]. Remote-controlled drug release via external stimuli such as light or magnetic fields further enhances therapeutic precision, aligning with point-of-care biosensors for real-time biomarker monitoring [136–138]. However, current systems face limitations in response speed and mechanical durability, as observed in pH/electric field-responsive chitosan-polyaniline hydrogels, which exhibit delayed activation cycles [139]. These challenges underscore the need for advanced material engineering to balance responsiveness with structural integrity in dynamic biological environments.

Synergistic therapeutic strategies are being explored to amplify treatment efficacy. Combining curcumin-loaded hydrogels with immune checkpoint inhibitors has shown potential to simultaneously target inflammatory pathways and tumor immune evasion mechanisms [140]. Nanocarrier-hydrogel hybrids, such as PEGylated nanogels, demonstrate pH/enzyme-triggered drug accumulation in colorectal cancer models, achieving tumor-specific delivery through hierarchical release mechanisms. This multi-scale approach capitalizes on the TME’s biochemical heterogeneity while mitigating off-target effects. Emerging platforms like G-quadruplex hydrogels further enable zero-order drug release and dual-functionality as biosensors, though their long-term biocompatibility requires rigorous validation [141].

Clinical translation faces multifaceted challenges that demand interdisciplinary solutions. Mechanistic ambiguities persist regarding hydrogel degradation rates in heterogeneous TMEs, necessitating advanced characterization techniques to map structure-function relationships. While chitosan-based hydrogels show promise, their inferior conductivity and fracture energy compared to native tissues limit practical applicability. Standardization of scalable manufacturing processes through 3D/4D bioprinting is critical for clinical-grade production, as demonstrated in shape-memory hydrogels with tunable porosity [142]. Environmental sustainability concerns, particularly for brain-targeted therapies, drive research into biodegradable formulations with minimal ecological footprint-a priority highlighted in recent lifecycle assessments of photoresponsive azobenzene-crosslinked hydrogels [143].

Future research directions should prioritize AI-powered predictive frameworks that integrate multi-omics data with patient-specific tumor profiles. Moving beyond static prediction, the concept of ‘Digital Twins’—virtual representations of a patient’s specific TME—could allow AI to simulate hydrogel degradation, drug distribution, and immune response in silico prior to implantation. This would transition the field from generalized formulation optimization to true personalized precision medicine. Furthermore, the integration of advanced biofabrication techniques like the “grafting-to” route for enzyme-nanogel synthesis holds significant promise. By covalently immobilizing therapeutic enzymes (e.g., catalase, superoxide dismutase) or epigenetic modulators within the hydrogel network, it is possible to create “living” drug depots that not only release drugs but also actively remodel the TME in real-time. For instance, a hydrogel co-loaded with curcumin and GPx via this route could sequentially or simultaneously execute drug delivery and redox regulation, offering a dynamic response to tumor heterogeneity [144]. Machine learning models capable of optimizing hydrogel porosity and crosslinking density could enable personalized material design, as suggested by recent successes in predicting drug release patterns using hybrid mechanistic-empirical models [134]. Advanced stimulus modalities, including wavelength-tunable NIR/UV activation systems, may improve tissue penetration depth-a limitation of current photoresponsive platforms. The development of hybrid biomaterials combining conductive polymers (e.g., PPy, PANI) with natural polymers via γ-radiation synthesis shows potential for creating smart implants, though radiation safety protocols require refinement [139]. The convergence of covalent “grafting-to” bioconjugation, multi-stimuli responsiveness, and AI-driven design could lead to the next generation of “intelligent” hydrogels capable of adaptive, feedback-controlled therapy. Ultimately, closing the gap between computational prediction and clinical validation through closed-loop AI systems will be crucial for realizing the full potential of intelligent hydrogel therapies in precision oncology.

## Conclusion

The integration of curcumin with hydrogel-based delivery systems constitutes a transformative strategy to surmount its profound pharmacokinetic limitations—including poor aqueous solubility, chemical instability, and rapid systemic metabolism—while concurrently amplifying its therapeutic efficacy in oncology. Hydrogels serve as an advanced platform where the encapsulation of curcumin within the 3D matrix or embedded nanocarriers provides a protective microenvironment, enhancing solubility and stability.

A pivotal advantage of this paradigm is the capacity for spatiotemporally controlled drug release, achievable through meticulous design of stimuli-responsive systems (e.g., pH, temperature, or enzyme-responsive). These “smart” hydrogels minimize off-target toxicity and enhance intratumoral accumulation by responding to specific pathological cues in the tumor microenvironment (TME). Furthermore, the platform’s potential is fully unlocked through combinatorial strategies, where the co-delivery of curcumin with chemotherapeutics, miRNAs, or photothermal agents generates potent synergistic effects. Ultimately, these hydrogel systems should not be viewed merely as passive carriers, but as active modulators that function as a ‘resensitization switch,’ capable of turning ‘cold’ or chemoresistant tumors into vulnerable targets for standard-of-care therapies. By concurrently modulating oncogenic signaling pathways, reversing multidrug resistance, and reshaping the immunosuppressive TME, curcumin-loaded hydrogels represent a promising frontier in the development of precision cancer therapies.

## Data Availability

No datasets were generated or analysed during the current study.
